# Predictors of delayed culture conversion among Ugandan patients

**DOI:** 10.1186/s12879-017-2335-7

**Published:** 2017-04-24

**Authors:** Daniel Atwine, Patrick Orikiriza, Ivan Taremwa, Arnold Ayebare, Suzan Logoose, Juliet Mwanga-Amumpaire, Amina Jindani, Maryline Bonnet

**Affiliations:** 1 Epicentre Mbarara Research Centre, PO box 1956, Mbarara, Uganda; 2 Mbarara University of Science and Technology, Mbarara, Uganda; 3 IRD UMI233 TransVIHMI-UM-INSERM U1175, Montpellier, France; 4 St. George’s, University of London, London, UK; 5 University of Montpellier 1, Montpellier, France

**Keywords:** Delayed culture conversion, Efficacy, HIV-negative TB patients, Time-to-detection, Treatment failure

## Abstract

**Background:**

Estimates of month-2 culture conversion, a proxy indicator of tuberculosis (TB) treatment efficacy in phase-2 trials can vary by culture-type and geographically with lower rates reported among African sites. The sub-study aimed at comparing TB detection rates of different culture media, within and across rifampicin-based regimens (R10, 15 and 20 mg/Kg) over a 6-month treatment follow-up period, and to establish predictors of month-2 culture non-conversion among HIV-negative TB patients enrolled at RIFATOX trial site in Uganda.

**Methods:**

Unlike in other Rifatox Trial sites, it is only in Uganda were Lowenstein-Jensen (LJ) and Mycobacteria growth indicator tube (MGIT) were used throughout 6-months for treatment monitoring. Conversion rates were compared at month-2, 4 and 6 across cultures and treatment-type. Binomial regression analysis performed for predictors of month-2 non-conversion.

**Results:**

Of the 100 enrolled patients, 45% had converted based on combined LJ and MGIT by month-2, with no significant differences across treatment arms, *p* = 0.721. LJ exhibited higher conversion rates than MGIT at month-2 (58.4% vs 56.0%, *p* = 0.0707) and month-4 (98.9% vs 88.4%, *p* = 0.0391) respectively, more so within the high-dose rifampicin arms. All patients had converted by month-6. Time-to-TB detection (TTD) on MGIT and social service jobs independently predict month-2 non-conversion.

**Conclusion:**

The month-2 culture conversion used in phase 2 clinical trials as surrogate marker of treatment efficacy is influenced by the culture method used for monitoring mycobacterial response to TB treatment. Therefore, multi-centric TB therapeutic trials using early efficacy endpoint should use the same culture method across sites. The Time-to-detection of MTB on MGIT prior to treatment and working in Social service jobs bear an increased risk of culture non-conversion at month-2.

**Trial registration:**

ISRCTN ISRCTN55670677. Registered 09th November 2010. Retrospectively registered.

**Electronic supplementary material:**

The online version of this article (doi:10.1186/s12879-017-2335-7) contains supplementary material, which is available to authorized users.

## Background

Culture conversion at month-2, is globally used as proxy indicator of tuberculosis (TB) chemotherapy efficacy in phase 2 clinical trials [1–3].

In addition to the treatment effect, several factors can affect the culture conversion at month-2, such as genetic polymorphism [4], age above 45 years, high pre-treatment sputum bacterial load, drug resistance extent of the radiographic involvement or presence of lung cavities, baseline time-to-detection (TTD) of TB, infection with W-Beijing genotype of *M. Tuberculosis*, smoking, and alcohol abuse [5–10]. Treatment interruption, irregularity in drug intake, and inadequate dosage, particularly of rifampicin may also lead to delayed culture conversion [11–13]. This may explain variability of culture conversion at month-2 across sites in multicenter clinical trials or amongst trials.

Although not much explored, the variations in culture conversion may also be influenced by type of culture media [3, 14]. Specifically, the solid culture medium (Löwenstein-Jensen [LJ]) tend to exhibit a slower growth and lower sensitivity as compared to liquid culture in a mycobacterium growth indicator tube (MGIT) [1, 15]. There is no clear guidance whether both solid and liquid based culture media should be used in tuberculosis clinical trials for outcome assessment. This is particularly important for some sites from high TB burden and limited resource countries which still do have limited access to liquid culture medium. Therefore, the impact of parallel use of different culture media within the same geographical patient population on the outcome evaluation of tuberculosis treatment regimen needs further assessment.

We present results from a sub-study of the Rifatox trial that evaluated the safety of high-dose rifampicin (R) (15 and 20 mg/Kg) administered during the first 4 months of HIV negative TB patients’ treatment in Uganda, Nepal and Bolivia [16].

This sub-study took advantage that within the Rifatox trial, the Ugandan site used both LJ and MGIT cultures for all patients during the 6 months treatment as per site guidelines unlike other international sites that used LJ alone and only up to month 2, so as to compare TB detection rates on different culture media, within and across rifampicin-based treatment regimens over a 6-month treatment follow-up period, and to establish predictors of month-2 culture non-conversion.

## Methods

### Study design

We conducted a secondary analysis of data of all 100 patients enrolled from the African site (Mbarara, Uganda) within the Rifatox Trial [16].

### Participants

The Mbarara site of the Rifatox trial was at Epicentre Mbarara Research Centre and was the only African site. The setting is endemic of TB and HIV. The trial consecutively recruited participants from the outpatient department (OPD) at Mbarara Regional Referral Hospital (MRRH).

Patients were eligible for enrolment in the trial if HIV negative, aged 18 years and above, with two sputum samples positive for tubercle bacilli on microscopy; having received less than a month of previous anti-tuberculosis chemotherapy; with a firm and accessible home address and if they consented to participation. Patients were excluded if they were critically ill, had extra-pulmonary TB, alcoholism, psychiatric illness, blood disorders, diabetes, epilepsy, HIV positivity, peripheral neuritis, pregnancy, a hemoglobin <7 g/dl, serum ALT levels >5 times the upper limit of normal (ULN), a creatinine clearance <30 ml/min, and rifampicin resistance.

### Study procedures

Socio-demographic characteristics specifically age, sex, district of residence, marital status and occupation, were collected using a standardized questionnaire which was administered in the local language. All patients underwent a physical examination by a medical doctor to record clinical data.

Patients were randomized to one of the 3 treatment regimens, that is: 1) Control Regimen (CR): 2 months of daily ethambutol (E) isoniazid (H) pyrazinamide (Z) Z and R at the usual dose of 10 mg/kg followed by 4 months of RH; 2) Study Regimen 1 (SR1):The regimen as above but with an increase in the dose of R to 15 mg/kg body weight daily for the first 4 months and a standard dose R (10 mg/kg) was given for the last 2 months; 3) Study Regimen 2 (SR2): The regimen as in SR1 but with an increase in the dose of R to 20 mg/kg body weight daily for the 4 months. Treatment was given under Direct Observation (DOT) by Domiciliary Treatment Monitor (DTM) or study nurse.

Patients provided spot sputum specimen at baseline, months 2, 4 and 6 for smear microscopy, LJ and manual MGIT (Becton, Dickinson, Franklin Lakes, NJ) cultures. Resistance testing was performed on decontaminated sample at baseline using GenoType MTBDRplus 2.0 (Hain Lifescience, Nehren).

In the laboratory, the specimens were decontaminated using the N-acetyl-L-cysteine and sodium hydroxide (1.5% final concentration). The decontaminated sputum was inoculated into two homemade LJ medium tubes and one MGIT tube. Negative culture results were reported after 56 days of incubation. Growth in the LJ and MGIT cultures, were checked for Acid fast bacilli and contamination using Ziehl-Neelsen (ZN) microscopy and blood agar culture respectively. ZN positive cultures were differentiated between *Mycobacterium tuberculosis (MTB)* and non-tuberculosis mycobacterium (NTM) using the SD TB Ag MPT64 Rapid system (SD Bioline, Kyongi-do, South Korea). Generally, the Time-to-positivity/detection was recorded for all TB patients using MGIT cultures.

Patients had weekly clinical assessment during first 2 months and then monthly with regular monitoring of liver function tests [16].

### Statistical analysis

Data were double-entered in a Voozanoo database (Epiconcept, Paris, France) and all statistical analysis was performed using Stata® software (v. 12, College Station, Texas, USA).

Relevant summary statistics were used to describe participants’ baseline characteristics. The proportion of patients with culture conversion out of those with prior baseline culture positivity was calculated at months-2, 4 and 6 by treatment regimen and by culture method (LJ, MGIT and combination of LJ + MGIT) after exclusion of patients with culture contaminated result from the denominator. Patients with contaminated samples were excluded in the subsequent analysis. Comparison of culture conversion between LJ and MGIT culture methods at each time point was performed using Mc’ Nemar exact test for matched data, while Pearson Chi2 was used to compare the culture conversion rates between different treatment regimens used. Our dependent variable was culture non-conversion at month-2, defined as: having a MTB growth on either LJ or MGIT culture at month-2. Independent variables used included: 1) baseline patients’ socio-demographics (age, sex, district of residence, marital status and occupation using definitions adapted from those used in the Uganda Demographic Health Survey reports [17]; 2) Baseline Clinical data, that is, body mass index (BMI) defined as low if <18.5 kg/m2 [18], sputum bacterial load using the World Health Organization (WHO) grading for LJ culture [19], time-to-detection of MTB on MGIT culture at baseline categorized into a binary variable using a cut-off of 14 days [20], hemoglobin level defined as low if <11 g/dL for males and ≤10.4 g/dl for females [21].

We fitted univariable and multivariable binomial regression models to establish the predictors of culture non-conversion at month-2. Variables associated with *P* < 0.4 in univariable analysis were included in the initial multivariable model after establishing the absence of multi-collinearity. The final model was systematically adjusted on treatment regimen, age, gender and baseline colony density. A 5% significance level was used. Tests for interaction and goodness-of-fit were performed.

## Results

**Fig. 1:**
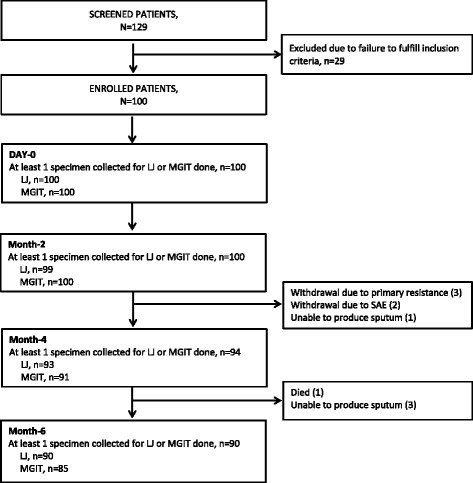
Study Profile

One hundred enrolled patients in Uganda had their sputum evaluated at different time points (Figure 1). The participants were predominantly males (80%) with a mean age of 36.2 years. They had a mean body weight of 51 kg and 44% were underweight (BMI < 18.5Kg/m^2^). Predominantly, patients had high bacillary loads of 2+ and above (87%) and high colony density of 3+ and above in LJ (80%). Overall, mean TTD of MTB on MGIT was 13.4 ± 10.6 days (Range: 3–51 days), with 65 out of 95 (68.4%) patients showing MTB growth within 14 days from MGIT inoculation at baseline. Majority of patients with baseline colony density of >2+ or ≤2+, had a TTD on MGIT of less than 14 days (79%) and above 14 days (72%), respectively, *p* < 0.0001. Three patients (3%) had isoniazid mono-resistance (Table 1).

**Table 1:** Participants’ baseline characteristics

Characteristic	Number	
Mean age in years (SD)	100	36.2 (11.6)
Age categories, n (%)		
18–24		20 (20.0)
25–49		64 (64.0)
> =50		16 (16.0)
Gender, male, n (%)	100	80 (80.0)
Marital status, n (%)	92	
Married		54 (58.7)
Separated/Divorced		8 (8.7)
Single/widowed		30 (32.6)
Occupation^a^, n (%)	92	
Peasant		16 (17.4)
Professional		9 (9.8)
Service		18 (19.6)
Unskilled manual		37 (40.2)
Student		6 (6.5)
Business		6 (6.5)
Transport mode to clinic, n (%)	91	
Motorbike		34 (37.4)
Car		16 (17.6)
Taxi		41 (45.1)
District of residence, n (%)	100	
Bushenyi		9 (9.0)
Isingiro		21 (21.0)
Mbarara		70 (70.0)
Relationship with Domiciliary treatment monitor, n (%)	97	
Parental family		42 (43.3)
Own family		22 (22.7)
Friends/in-laws		33 (34.0)
Current smoker, n (%)	89	35 (39.3)
TB treatment regimen, n (%)	100	
Standard regimen (R10mg/kg)		33 (33.0)
Regimen 1 (R15mg/kg)		33 (33.0)
Regimen 2 (R20mg/kg)		34 (34.0)
Clinical Parameters		
Mean body weight in kg (SD)	100	51.2 (7.8)
Mean BMI in Kg/m^2^ (SD)	99	19.0 (2.4)
BMI <18.5, n (%)		44 (44.4)
Biochemical parameters		
Mean hemoglobin in g/dl (SD)	100	11.7 (1.8)
Hemoglobin grade^b^		
Mild anemia, n (%)		13 (13.0)
Moderate anemia, n (%)		11 (11.0)
Severe anemia, n (%)		6 (6.0)
Biological Parameters		
Baseline smear positivity, n (%)	100	100 (100.0)
Scanty		4 (4.0)
1+		9 (9.0)
2+		25 (25.0)
3+		62 (62.0)
Baseline LJ positivity, n (%)	100	98 (98.0)
Colony density, n (%)		
Negative		2 (2.0)
1+		4 (4.0)
2+		14 (14.0)
3+		56 (56.0)
4+		24 (24.0)
Baseline MGIT positivity, n (%)	100	95 (95.0)
Baseline combined LJ/MGIT positivity, n (%)	100	100 (100.0)
Mean Time-to-detection (TDD) of MTB on MGIT in days (±SD)	95	13.4 ± 10.6
TDD of MTB on MGIT in days, <14 days, n (%)	95	65 (68.4)
Primary Isoniazid resistance, n (%)	100	3 (3.0)

*SD* standard deviation, *BMI* body mass index, *LJ* Löwenstein-Jensen, *MGIT*, mycobacterium growth indicator tube, *MTB* mycobacterium tuberculosis complex

^a^Occupation: 1) Peasant represents people earning from agriculture; 2) professional represents those working in certified, managerial or technical jobs; 3) Social service representing mainly transport services like motor-bike riders and those in unskilled manual (like bar/restaurant attendants, house maids, truck attendants); 4) Students representing those still in school and not working; 5) Business representing those involved in their own businesses. ^b^Hemoglobin: Mild (male:10–10.9 g/dl, Female: 9.5–10.4), Moderate (Male: 9.0- < 10.0, Female: 8.5- < 9.5), Severe (Male:7.0- < 9.0, Female: 6.5- < 8.5based on Division of AIDS (DAIDS) guideline

Overall, using both LJ and MGIT, 45% had converted on culture by month-2. Culture conversion was 50%, 40.6% and 44.1% for patients in the 10, 15 and 20 mg/Kg rifampicin arms, respectively, *p* = 0.721 (Table 2). It increased to 91.2%, 83.3% and 86.7% at month-4 (*p* = 0.685). All patients were culture negative at month-6.

**Table 2:** Culture conversion by culture method and treatment arm at month-2, 4 and 6

Month	Culture	Overall	Treatment Arm			
			CR	SR1	SR2	*p*-value^b^
		Converted, n/N, %	Converted, n/N, %	Converted, n/N,%	Converted, n/N, %	
Month-2	LJ	51/91 (56.0)	17/32 (53.1)	14/27 (51.9)	20/32 (62.5)	0.697
	MGIT	46/95 (48.4)	18/33 (54.6)	13/31 (41.9)	15/31 (48.4)	0.601
	% difference	7.6	−1.5	10	14.1	
	*p*-value^a^	0.0707	1.0000	0.1250	0.2891	
	Both LJ and MGIT	45/100 (45.0)	17/34 (50.0)	13/32 (40.6)	15/34 (44.1)	0.721
Month-4	LJ	88/89 (98.9)	31/31 (100.0)	28/29 (96.6)	29/29 (100.0)	0.652
	MGIT	76/86 (88.4)	29/32 (90.6)	24/27 (88.9)	23/27 (85.2)	0.913
	% difference	10.5	9.4	7.7	14.8	
	*p*-value^a^	0.0391	0.5000	0.6250	0.2500	
	Both LJ and MGIT	82/94 (87.2)	31/34 (91.2)	25/30 (83.3)	26/30 (86.7)	0.685
Month-6	LJ	87/87 (100.0)	33/33 (100.0)	26/26 (100.0)	28/28 (100.0)	NA
	MGIT	78/78 (100.0)	29/29 (100.0)	26/26 (100.0)	23/23 (100.0)	NA
	% difference	0.0	0.0	0.0	0.0	
	*p*-value^a^	1.0000	1.0000	1.0000	1.0000	
	Both LJ and MGIT	88/88 (100.0)	34/34 (100.0)	26/26 (100.0)	28/28 (100.0)	NA

^a^Exact McNemar *p*-value

^b^Fishers’ exact *p*-value

*Converted* culture converted, *CR* control regimen (10 mg/kg rifampicin), *SR1* study regimen1 (15 mg/kg rifampicin), *SR2* study regimen2 (20 mg/kg rifampicin), *LJ* Löwenstein-Jensen, *MGIT* mycobacterium growth indicator tube

Contamination was reported in 5/99 (5.1%), 2/93 (2.2%) and 0/90 (0%) of the patients with LJ done at month-2, 4 and 6 respectively. On the other-hand, contamination was reported in 3/85 (3.5%) patients with MGIT performed at month-6. No MGIT contamination was noted before month 6 follow-up. None of the patients had contamination on both LJ and MGIT at the same visit.

The overall estimates of culture conversion by LJ were higher than with MGIT at months 2 (56.0% vs 48.4%, *p* = 0.0707) and month-4 (98.9% vs 88.4%, *p* = 0.0391) (Table2). Although none showed statistical significance the differences in conversion rates between LJ and MGIT at month-2 seem more higher within patients in high-dose rifampicin arms (10% for 15 mg/Kg and 14.1% for 20 mg/Kg) than patients in the control arm (−1.5%).

TDD of MTB on baseline MGIT culture and occupation were significantly associated with the culture non-conversion at month-2 after adjusting for age, gender, treatment regimen, and baseline colon density. Patients with baseline TTD below 14 days had a 2-fold increased risk of month-2 non-conversion as compared to those with TTD of 14 days and above, (aRR = 2.1, [95% CI: [1.18–3.93], *p* = 0.013). On the other-hand, within occupations, patients working in “social service jobs”, which in this study accounts mostly for motor-bike passenger riders, were significantly associated with a 3-fold increased risk of month-2 culture non-conversion (RR = 3.0, [95% CI:1.11–8.19], *p* = 0.031) as compared to those in formal professional jobs (Table 3).

**Table 3:** Results of univariable and multivariable binomial regression analysis of predictors of month-two culture non-conversion

Characteristic	Converted, n (%)	Non-converted, n (%)	Unadjusted RR [95% CI]	Adjusted RR [95% CI]
Age in years, *n* = 100				
18–45	36 (43.4)	47 (56.6)	1.2 [0.70–2.06]	0.6 [0.32–1.32]
> 45	9 (52.9)	8 (47.1)	1.0	1.0
Gender, *n* = 100				
Female	10 (50.0)	10 (50.0)	1.0	1.0
Male	35 (43.8)	45 (56.3)	1.1 [0.70–1.82]	1.1 [0.60–2.06]
Marital status, *n* = 92				
Married	26 (48.2)	28 (51.9)	1.0 [0.67–1.61]	
Separated/Divorced	2 (25.0)	6 (75.0)	1.5 [0.88–2.57]	
Single/widowed	15 (50.0)	15 (50.0)	1.0	
Occupation, *n* = 92				
Peasant	11 (68.8)	5 (31.3)	0.9 [0.29–3.04]	0.8 [0.26–2.65]
Professional	6 (66.7)	3 (33.3)	1.0	1.0
Social service	3 (16.7)	15 (83.3)	2.5 [0.97–6.44]	3.0 [1.10–8.19]
Unskilled manual	17 (46.0)	20 (54.1)	1.6 [0.61–4.28]	^a^1.5 [0.56–3.78]
Student	3 (50.0)	3 (50.0)	1.5 [0.44–5.09]	2.1 [0.61–7.11]
Business	3 (50.0)	3 (50.0)	1.5 [0.44–5.09]	1.3 [0.40–4.36]
District of residence, *N* = 100				
Bushenyi	5 (55.6)	4 (44.4)	1.0	
Isingiro	8 (38.1)	13 (61.9)	1.4 [0.62–3.11]	
Mbarara	32 (45.7)	38 (54.3)	1.2 [0.57–2.62]	
Current Smoker, *n* = 89	15 (42.9)	20 (57.1)	1.1 [0.74–1.62]	
TB treatment regimen, *n* = 100				
Standard regimen	17 (50.0)	17 (50.0)	1.0	1.0
Regimen 1	13 (40.6)	19 (59.4)	1.5 [0.55–3.87]	0.8 [0.45–1.34]
Regimen 2	15 (44.1)	19 (55.9)	1.3 [0.49–3.29]	1.1 [0.69–1.84]
Body mass index in kg/m2, *n* = 99				
18.5 and above	25 (45.5)	30 (54.6)	1.0	
< 18.5	20 (45.5)	24 (54.6)	1.0 [0.70–1.44]	
Colony density at baseline, *n* = 98				
1+	2 (50.0)	2 (50.0)	1.0	1.0
2+	8 (57.1)	6 (42.9)	0.9 [0.27–2.71]	0.5 [0.12–2.01]
3+	23 (41.1)	33 (58.9)	1.2 [0.43–3.22]	0.5 [0.11–1.82]
4+	11 (45.8)	13 (54.2)	1.1 [0.38–3.09]	0.4 [0.09–1.44]
Time-to-detection (TDD) of MTB on MGIT at baseline				
Less than 14 days	24 (36.9)	41 (63.1)	1.6 [0.98–2.54]	2.1 [1.18–3.93]^b^
14 days and above	18 (60.0)	12 (40.0)	1.0	1.0
Hemoglobin levels, *n* = 100				
Normal	28 (40.0)	42 (60.0)	1.0	
Low	17 (56.7)	13 (43.3)	0.7 [0.46–1.13]	
Missed atleast a dose in first 2 months, *n* = 100	3 (23.1)	10 (76.9)	1.5 [1.04–2.13]	

Multivariable model characteristics: *N* = 87, deviance = 100.63, Binomial model-based goodness of fit, *p* = 0.0177, Logit model-based goodness of fit, *p* = 0.5291

*RR* crude risk ratio, *aRR* adjusted risk ratio, *95% CI* 95% Confidence Interval, *LJ* Löwenstein-Jensen, *MGIT* mycobacterium growth indicator tube, *NA* not applicable, *MTB* mycobacterium tuberculosis ^a^ *p* = 0.031; ^b^ *p* = 0.013

## Discussion

This sub-analysis of the data from Ugandan site within the Rifatox trial, shows that the low culture conversion at month-2 was due to delayed culture conversion and not treatment failure. Indeed, 87.2% of patients converted by month-4 and 100% by month-6 without initiation of second-line treatment. Culture non-conversion at month-2 suffers a lower specificity in predicting TB relapse or treatment failure [22] a phenomenon partially observed in our study given that all month-2 non-converters finally converted by month 6. No significant differences were observed in the conversion rates across 10, 15 and 20 mg/kg rifampicin-based regimens using LJ, MGIT or combination of LJ and MGIT. Despite the low sample size on which this sub-analysis was performed, these results are consistent with what was reported in the Rifatox trial using LJ culture only [16]. However, given the good safety results of high-dose rifampicin shown in two recent trials [16, 23] and the difficulty to rely on month-2 culture conversion as surrogate endpoint of treatment efficacy [24–26], a phase3 efficacy trial of 4 month regimens based on high-dose rifampicin is under implementation. This Rifashort trial (NCT02581527) will evaluate 1200 mg and 1800 mg rifampicin daily in the reduction of treatment duration for pulmonary tuberculosis from 6 months to 4 months.

The observed combined LJ/MGIT month-2 culture conversion rate (45%), was lower than that reported (61%) in another trial that involved patients in Central Uganda [14]. The slow conversion rates may partly be explained by the fact that majority of the patients (>80%) had high baseline bacillary load and colony density. The absence of association between baseline sputum bacterial load and the absence of culture conversion at Month 2 is possibly hidden by the selection of smear-positive patients and by a very high proportion of patients with high bacterial load overall. This is a reflection of late medical consultation or delayed TB diagnosis among TB patients. Delayed culture conversion may potentially elongate patients’ period of infectiousness, a challenge in TB control efforts. Unfortunately, since patients were not followed up after completion of treatment in the Rifatox phase2 trial, it was not possible to report on whether delayed sputum converters also had a higher risk of relapse than early converters. On a positive side, this will also be assessed within the Rifashort trial.

Our study noted a 2-fold increase in risk of culture non-conversion at month-2 in patients with baseline TTD of MTB on MGIT below 14 days as compared to those with TTD of 14 days or above [10]. This difference may partially be due to the high bacillary colony density prior to treatment (80% > 2+). However, it is interesting to note that the significant association remained after adjustment on baseline colony density. On the other-hand, baseline colony density showed no significant association with culture non-conversion at month-2, a finding that could be explained by an overwhelmingly higher proportion of patients with high colony density (>80%) in this study. The 3-fold increase in risk of culture non-conversion at month-2 noted in our study among patients employed in social service jobs (mainly commercial motor-bike passenger riders) as compared to those in formal professional employment, shows the role of social factors and the need for their consideration within TB treatment efficacy trials. The Motor-bike passenger riders have a great social sphere of interaction, and may play a key role in TB transmission within urban and semi-urban settings. Further exploration into the reasons underlying the increased vulnerability of this population group is needed.

This sub-study confirms the slow bacteriological response to treatment or the presence of persister bacilli populations reported among African patients, something that has complicated the estimation of early TB chemotherapy efficacy in a number of multi-centric clinical trials [1, 2, 16, 27]. Indeed, this delayed conversion among Ugandan patients, offers more evidence to the suggestion made in the retrospective study of several phase 3 TB trials conducted by the British Medical Council in Hong Kong and East Africa, recommending the use of month-3 instead of month-2 culture conversion endpoint as a surrogate of treatment efficacy within the phase II East African trials [26].

As expected, LJ which is known to be a less sensitive culture method than MGIT had a 10% higher month-2 culture conversion rate as compared to MGIT. The significantly high overall difference in conversion rates between LJ and MGIT especially at month4, only confirms the declining sensitivity of LJ in detecting TB with declining bacillary loads during treatment follow-up, a finding also consistent with other studies [1, 15, 28]. It highlights the necessity to consistently use the same culture methods for early treatment efficacy endpoints across sites within a TB clinical trial. This approach, will not affect the comparisons of the conversion rates between treatment arms. Likewise, culture conversion rates across clinical trials can easily be compared or pooled as long as they are based on the same culture method. Also, our results do not support the need to combine LJ and MGIT cultures in the same trial for the microbiological outcome assessment. However, this would require to be further assessed in a larger study. Surprisingly, this effect of culture media used, seem higher and faster in the high rifampicin dose groups, with bigger differences in TB conversion rates between LJ and MGIT manifesting as earlier as month-2 of treatment follow-up, a phenomenon that may perhaps be explained by a faster reduction in LJ sensitivity following a more rapid decline in bacilli population due to higher doses of rifampicin [13].

The study has several limitations: i) Since it was a sub-study from the Ugandan site only, numbers are very small. The small sample size reduced the power to show a difference of culture conversion between arms or between culture methods and may have hindered significance of some predictor variables of non-conversion. This also may have contributed to the high deviance and inadequate goodness of fit seen with the multivariable binomial regression model (*p* = 0.0177), although a good fit was achieved on a re-run of the final model with a logit-based model (*p* = 0.5291). ii) Our study being limited to only adult HIV negative TB patient population limits its generalizability to the HIV positive population. Similarly, the study enrollment was limited to smear-positive patients and the results cannot be generalized to smear-negative culture positive TB patients. Also, being a sub-analysis within a single site, findings cannot be generalized to other sites from different regions. Iii) Some variables like chest radiological findings were not collected within the Rifatox trial and could not be explored in the analysis of predictors of non-conversion. iv) Finally, because the Rifatox trial was a phase 2 safety trial, post treatment culture follow-up was not part of the trial procedures and so, we could not establish whether the delayed converters had a higher relapse rate than early converters.

## Conclusions

The month-2 culture conversion used in phase 2 clinical trials as surrogate marker of treatment efficacy is influenced by the culture method used for monitoring mycobacterial response to TB treatment. Therefore, multi-centric TB therapeutic trials using early efficacy endpoint should use the same culture method across sites. The Time-to-detection of MTB on MGIT prior to treatment initiation and working in Social service jobs bear an increased risk of culture non-conversion at month-2.

## Additional file

Additional file 1: Dataset. (CSV 14.4kb)

## Abbreviations

ALT: Alanine aminotransferase
BMI: Body mass index
DOT: Directly observed treatment
DTM: Domiciliary treatment monitor
E: Ethambutol
HIV: Human immunodeficiency virus
H: Isoniazid
LJ: Lowenstein-Jensen
MGIT: Mycobacteria growth indicator tube
NTM: Non-tuberculosis mycobacterium
Z: Pyrazinamide
RH: Rifampicin and isoniazid
R: Rifampicin
RR: Risk ratio
SR1: Study regimen one
SR2: Study regimen two
TB: Tuberculosis
ULN: Upper limit of normal
